# Some Agreement on Kin Selection and Eusociality?

**DOI:** 10.1371/journal.pbio.1002133

**Published:** 2015-04-24

**Authors:** David C. Queller, Stephen Rong, Xiaoyun Liao

**Affiliations:** 1 Biology Department, Washington University in St. Louis, St. Louis, Missouri, United States of America; 2 Department of Ecology and Evolutionary Biology, Rice University, Houston, Texas, United States of America

## Abstract

The authors of "Relatedness, Conflict, and the Evolution of Eusociality" respond to objections raised by Martin Nowak and Benjamin Allen.

Our paper [1] was not about the exact mathematical equivalence of inclusive fitness and other approaches. Theoreticians will continue to debate this question, but the rest of us want to know whether it matters for biology. We asked whether the model of Nowak, Tarnita, and Wilson (NTW)[2], when applied to their chosen test case of eusociality, makes any important difference. Does it refute kin selection theory? Does it offer new insights? The answer to both questions is no.

Now Nowak and Allen [3] suggest that we have misinterpreted NTW. For example, NTW did not mean that relatedness is unimportant. Instead, they only meant that if relatedness is high and held constant, other factors determine which species evolve eusociality, and that this is an issue the kin selectionists have not considered. On the contrary, it is completely obvious from Hamilton’s rule; if you hold relatedness constant, differences will be determined by variation in costs and benefits. There have also been more specific studies about synergistic factors affecting these costs and benefits [4,5]. Moreover, if this is the basis for NTW’s claim that relatedness is not causal, then we have shown that NTW’s other parameters are also not causal, because when we force them to be constant, only variation in relatedness matters [1]. Finally, this apparent concession about the importance of relatedness is perplexing, given that Nowak and Allen expend significant effort questioning the details of exactly how we modeled lower relatedness, while continuing to equivocate about the real issue of how relatedness matters. Low relatedness groups are real and can be formed in many ways, but with offspring control they do not give rise to eusociality [6]. If Nowak and Allen think otherwise and believe that there are reasonable ways to lower relatedness so that it does not make eusociality harder to evolve, then they should show how.

We could direct similar skepticism at Nowak and Allen’s [3] interpretations of the other two NTW claims that we investigated. But let us accept at face value all three of their interpretations about what NTW meant. If NTW did not actually mean that relatedness is unimportant, and if they did not mean that workers are merely robotic extra-somatic projections of the queen’s genome, and if they did not mean that eusociality was as hard to evolve as suggested in their main examples, then we are in happy agreement! But if this is so, why do they not just explicitly say, for example, “our method agrees with inclusive fitness in showing that higher relatedness is crucial in the evolution of eusociality”? Perhaps because it would require admitting that what we have learned about eusociality from kin selection models still stands, and that the NTW models, despite their much greater complexity, have so far added little more.
